# XSSJS inhibits hepatic fibrosis by promoting the miR-29b-3p/VEGFA axis *in vitro* and *in vivo*

**DOI:** 10.1042/BSR20212241

**Published:** 2022-02-25

**Authors:** Tianyao Zhang, Yu Yang, Baojia Wang, Long Wang, Dong Wang, Ning Cao, Jinyu Shi

**Affiliations:** 1 Department of Fundamental Medical Science, Chengdu University of Traditional Chinese Medicine, Chengdu, Sichuan, China; 2 Department of Fundamental Medical Science, Shaanxi University of Traditional Chinese Medicine, Xianyang, Shaanxi, China

**Keywords:** hepatic fibrosis(HF), miR-29b-3p, pathological angiogensis, VEGFA, Xueshisanjiasan (XSSJS)

## Abstract

Hepatic pathological angiogenesis (HPA) is the key event of hepatic fibrosis (HF). Xueshisanjia powder (XSSJS), a Chinese herbal compound, is beneficial for alleviating pathological angiogenesis of hepatic tissue. The present study attempts to reveal the effect and mechanism of XSSJS via regulating miR-29b-3p/VEGFA axis against pathological angiogenesis in HF. In *in vitro* model, human embryonic kidney 293T cells were transfected with miR-29b-3p mimics, whereby the expression of miR-29b-3p was tested by real-time quantitative polymerase chain reaction (RT-qPCR), ensued by Luciferase assay determining the relationship between miR-29b-3p and vascular endothelial cell growth factor A (VEGFA). In addition, miR-29b-3p mimic transfected into the activated hepatic stellate cell T6 (HSC-T6). The Cell-Counting-Kit 8 (CCK8) and 5-Bromodeoxyuridine (BrdU) staining were first utilized to detect the antiproliferative efficiency of XSSJS following the XSSJS compound serum intervention, and then used to observe the expression of transforming growth factor-β (TGF-β), VEGFA, platelet-derived growth factor (PDGF) via RT-PCR, Western blot (WB), and Immunofluorescence (IF) methods. During the * in vivo* model, XSSJS with boil-free granules were fed to Wistar rats with liver fibrosis caused by intraperitoneal injection of pig serum followed by the transfection of miR-29b-3p adeno-associated virus (AAV). Hematoxylin–Eosin (HE) staining was used for histopathology assessment. The expression of miR-29b-3p, VEGFA, PDGF, TGF-β have been investigated in liver tissue using RT-PCR, WB, IF. The results verified that XSSJS could up-regulate miR-29b-3p and suppress the expression of VEGFA, PDGA, and TGF-β. In mechanism, miR-29b-3p primarily targeted the 3′UTR of VEGFA. In conclusion, XSSJS could modulate miR-29b-3p/VEGFA axis to inhibit the pathological angiogenesis of HF.

## Introduction

**Figure 1:**
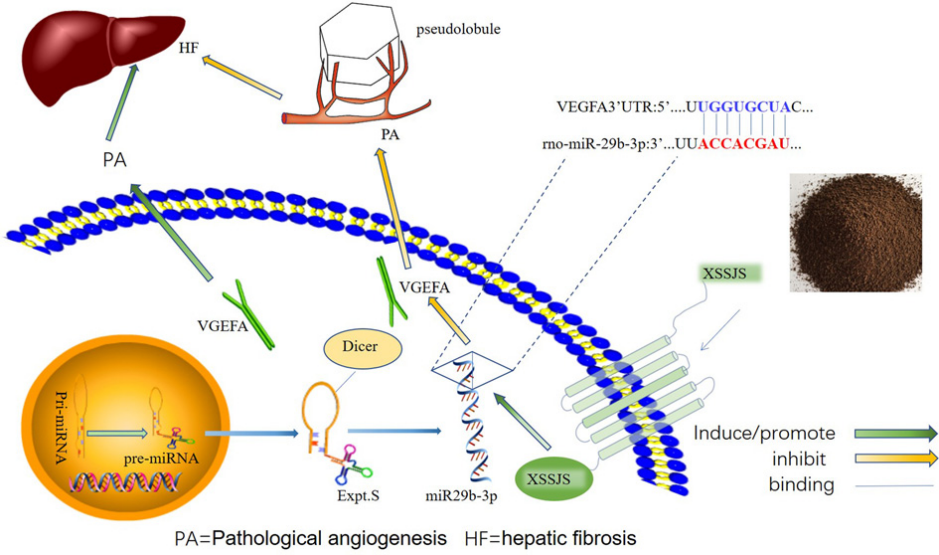
A schematic diagram with XSSJS promoting miR-29b-3p/VEGFA axis The schematic diagram representing the subject of the article shows that the XSSJS could down-regulate the expression of VEGFA and inhibit the PA of HF.

Hepatic fibrosis (HF) is a common pathological hallmark of chronic liver diseases (CLDs) [1]. Hepatic pathological angiogenesis (HPA) associates strong implication in the development of HF, which is recognized as a central event in hepatic stellate cells (HSCs) activation [2,3], inflammatory response [4–6] and hepatic vascular resistance (IHVR) [7,8]. In the progression of HPA, vascular endothelial growth factor (VEGFA) contributes to the proliferation, migration, differentiation of vascular endothelial cells and the formation of vascular lumen [9–11], which eventually leads to intrahepatic angiogenesis and hepatic sinusoidal capillarization [12,13]. Therefore, it is of great significance to restrain pathological angiogenesis for liver disease deterioration, especially targeting the excessive VEGF [14,15]. However, it is already known that the western drug of anti-angiogenesis such as Sorafenib, Bevacizumab and Sunitinib, present the obvious adverse effects, and were forced to interrupt treatment [16–18]. Noticeably, accumulated evidence have demonstrated that the use of traditional Chinese medicines (TCMs) have increased worldwide due to their exclusive properties, that activates blood circulation to dissipate stasis, which could prevent liver fibrosis via reducing liver tissue inflammation [19,20] and inhibiting pathological angiogenesis [21,22]. From TCM perspective, the pathology of liver fibrosis is the obstruction of collaterals by phlegm and blood stasis, so the therapy relies on its virtue of clearing phlegm, assisting blood circulation and dredging the collateral vessels. The extensive use of Xueshisanjia (XSSJS) for clinical treatment of CLD is owing to its significant clinical efficacy and inherent virtue of removing blood stasis and clearing the collateral vessels. Modern studies have shown that the anti-HF mechanisms of XSSJS include the inhibition of rat HSC-T6I, III collagen biosynthesis [23], as well as the pathological angiogenesis, accompanied by reducing microvascular density in fibrotic liver tissue and promoting the expression of VEGFA mRNA and protein [24–26]. Since, microRNAs are enriched in the liver tissue and involved in regulating the physiological and pathological processes, miR-29b is particularly expressed 100-times higher in HSCs than in other liver parenchymal cells [27]. miR-29b-3p is affiliated to miR-29b family, which bears high in-group similarity of mature sequences for humans, rats, and mice [28,29]. Some studies indicated that the overexpression of miR-29b is inversely proportional to the occurrence of liver fibrosis [30], and the main mechanism of miR-29b-3p to ease liver fibrosis is to inhibit the activation of HSCs [31,32], thus up-regulating miR-29b-3p is an effective strategy against HF. Previous researchers have been mostly absorbed in studying the physiological roles of miR-29b-3p on HF, yielding a general absence of the report on the mechanisms or target genes that cause the PA phenotypes. Therefore, we interest ourselves in the therapeutic effect and mechanisms of XSSJS on anti-fibrosis by regulating with miR-29b-3p/VEGFA axis, and the schematic presentation of the XSSJS inhibit PA via regulating miR-29b-3p/VEGFA axis is shown in Figure 1.

## Materials and methods

### Cell cultured and modeling

Human embryonic kidney cells (HEK-293T cells) were acquired from Procell Biotech Solutions Co. Ltd. (Shanghai, China), and cultured with 55% DMEM+40% fetal bovine serum (FBS)+5% DMSO. The rat hepatic stellate cell beads (HSCs-T6), obtained from Fusheng Industrial Co., Ltd (Shanghai, China), were cultured with DMEM*+*10% FBS+1% (penicillin/streptomycin solution). Transforming growth factor-β1 (TGF-β1) were purchased from PeproTech China (Suzhou, China), when HSC-T6 was proliferating at logarithmic rate, the digestion of cells was achieved with 0.25% trypsin, and 10^5^ cells/well were inoculated in 96-well plates after counting, TGF-β1 (10 ng/ml) was instilled in the model wells but not in those control ones. After 24 h, the supernatants and cells were subject to collection.

### Reagents and antibodies

TRIzol reagent was purchased from Invitrogen (Invitrogen, U.S.A.). The miR-29b-3p mimics and the negative control vectors (miR-29b-3p mimics NC) were synthesized and designed by Integrated Biotech Solutions Co. Ltd. (Shanghai, China IBS-01). U6 serves as an internal reference for miR-29b-3p and its sequence is shown in Table 1. The sequence of rno-miR-29b-3p is 3′ uugugacuaaaguuuaccacgau, and NCBI Gene ID is 104797378. VEGFA-overexpressing plasmid and the negative control vectors were synthesized and designed by SanGon Biotech Co., Ltd (Shanghai, China), which gene sequence is VEGFA 3′UTR (1634–1641): 5′ aucauuuauuuauuggugcuac, and NCBI Gene ID is 83785. Lipofectamine™ 2000 transfection reagent (Invitrogen, Thermo Fisher Scentific, China) was applied for cell transfection. The miR-29b-3p adeno-associated virus (AAV) and the negative control vectors (the miR-29b-3p NC AAV) were designed and synthesized from SanGon Biotech Co., Ltd (Shanghai, China). The primary antibodies, such as anti-VEGFA (bs-1665R, Rabbit, 1:1000), anti-TGF-β1 (bs-0086, Rabbit, 1:1000) were bought from Boaosen Biotechnology Co., Ltd (Beijing, China); anti-platelet-derived growth factor (PDGF; Gb-11261, Rabbit, 1:1000) were purchased from Xavier Biotechnology Co., Ltd (Wuhan, China); anti-CD31 (platelet endothelial cell adhesion molecule-1; 130 kDa, Rabbit, 1:1000), anti-CD34 (Gb-4000, Rabbit, 1:1000) and anti-β-Actin (Gb13001-1, Rabbit, 1:1000) were bought from Wuhan Google Biotechnology Co., Ltd (Wuhan, China); anti-glyceraldehyde-3-phosphate dehydrogenase (GAPDH; Mab5465, Mouse, 1:2000) were bought from Multi Sciences Biotechnology Co., Ltd (Hangzhou, China); dual-luciferase gene kit was bought from Beyotime Biotechnology Co., Ltd (Shanghai, China); luc-3′-UTR-WT/MUT were designed by SanGon Biotech Co., Ltd (Shanghai, China). Cell-Counting-Kit-8 (CCK8) and 5-Bromodeoxyuridine (BrdU) detection reagents were purchased from SanGon Biotech Co., Ltd (Shanghai, China).

**Table 1:** The primer sequences used in real-time quantitative polymerase chain reaction analysis

Gene	Primer	Sequences (5′–3′)
*R-Actin*	R-Actin-F	TGTCACCAACTGGGACGATA
	R-Actin-R	GGGGTGTTGAAGGTCTCAAA
*R-TGF-β*	R-TGF-β-F	GACCGCAACAACGCAATCTATGAC
	R-TGF-β-R	CTGGCACTGCTTCCCGAATGTC
*R-VEGFA*	R-VEGFA-F	CACGACAGAAGGGGAGCAGAAAG
	R-VEGFA-R	GGCACACAGGACGGCTTGAAG
*R-PDGF*	R-PDGF-F	TCTCTGCTGCTACCTGCGTCTG
	R-PDGF-R	AAGGAGCGGATGGAGTGGTCAC
*U6*	Sn-U6-F	CTCGCTTCGGCAGCACA
	Sn-U6-R	AACGCTTCACGAATTTGCGT
*miR-29b-3p*	mi-29b-F	CGCGTAGCACCATTTGAAATC
	mi-29b-R	AGTGCAGGGTCCGAGGTATT
*RT-primer*	GTCGTATCCAGTGCAGGGTCCGAGGTATTCGCACTGGATACGACAACACT	

### miR-29b-3p mimics transfection and XSSJS intervention *in vitro*

To overexpress the miR-29b-3p gene, HEK-293T cells were transfected with miR-29b-3p mimic and miR-29b-3p inhibitor via Lipofectamine 2000 transfection reagent (Invitrogen, Thermo Fisher Scentific, China) with reference to the instructions of the manufacturer. Real-time PCR analysis was employed to assess the expression of miR-29b-3p for 48 h of cell transfection. Thereafter, the mechanism of miR-29b-3p and VEGFA on the molecules via luciferase assay was confirmed. The target promoter fragment was cloned from genomic DNA employed PCR technology and inserted into luciferase reporter gene plasmid PGL3-BASIC by double digestion assay of restriction enzyme SacI and XbaI, the luciferase activity of VEGFA-WT was tested following 24 h of cell con-transfection with mimic-miR-29b-3p and VEGFA plasmids (firefly luciferase was used as a reporter gene and *Renilla* kidney luciferase was used as an internal reference gene). In order to further reveal the regulatory relationship between miR-29b-3p/VEGFA axis and XSSJS intervention in HF, and XSSJS containing serum was used for intervention following being transfected with an miR-29b-3p mimic and miR-29b-3p mimic NC in activated HSCs [33]. Except for the control and model groups, which were transfection-free, the miR29b-3P group and XSSJS+miR-29b-3p group were transfected with the miR-29b-3p mimics; miR-29b-3p NC group and XSSJS+ miR-29b-3p NC group were transfected with the miR-29b-3p mimics NC (negative control vectors) according to the instructions of the manufacturer and relative reference [34]. The transfection efficiency was confirmed by RT-PCR after 24 h.

### Cell proliferation efficiency analysis

Cellular proliferation was assessed using CCK8 and BrdU cell proliferation detection kits. Following the activation of HSC-T6 were the transfection with miR-29b-3p mimics and mimic NC, the cells were digested with 0.25% trypsin following HSC-T6’s logarithmic period, and 10^5^ cells/well were inoculated in 96-well plates after counting and were washed three-times in PBS. Twelve hours later, the original medium was changed to the medium with XSSJS containing serum when fusion rate of the cells reaches 80–95%. After 24 h of culturing, the biocompatibility and cytotoxicity of HSCs-T6 were assayed using a CCK8. According to the manufacturer’s instructions, 10 μl CCK8 reagent was administered to each well before harvesting, and the optical densities (OD) value at 450 nm was measured after 4 h of incubation. Each experiment was repeated three times.

Cell proliferation was also assessed by the number of nuclei that were 4′,6-diamidino-2-phenylindole (DAPI) stained and marked with BrdU. During HSC-T6’s in logarithmic period, BrdU with concentration of 20 μmol/ml was added into the culture medium. The secondary antibody for BrdU was labeled TRITC. Images were obtained using an optical microscope (400×; Olympus Corporation, Tokyo, Japan).

### XSSJS no-decoction granule prescription

XSSJS no-decoction granules contained turtle shell 15 g, pangolin 3 g, batryticated silkworm 10 g, ground beetle 10 g, peach kernel 10 g, bupleurum 5 g (total 53g). The same batch was purchased from Sichuan New green Pharmaceutical Science and Technology Development Co. Ltd. The Qualification Certificate No.: 15013726.

### Experimental animals

Adult (8–10 weeks old) male Wistar rats weighing 180 ± 20 g were obtained from Chengdu Dasuo Biotechnology Co., Ltd (License No.: SCXK(Sichuan), 2013-24, Certificate No.: 0002715). All animal experiments took place at Experimental Animal Center of Chengdu University of Traditional Chinese Medicine. A total of 80 Wistar rats were accommodated under a temperature-controlled condition (22 ± 2°C). Sixty rats were randomly and evenly spread into six groups (*n*=10 each) including control group, model group, miR-29b-3p group, miR-29b-3p NC group, XSSJS+miR29b-3P group, XSSJS+miR-29b-3p NC group. All of the animals were provided with day/night shift every 12 h, and unlimited access to water and rodent chow.

### Models, transfection and specimen collection *in vivo*

After a week of routine feeding, except for the control group via gavage with the same volume of 9 g/l normal saline, the remaining groups were fed with XSSJS granule prescription once a day on a regular time basis for 22 weeks. XSSJS granules prescription were dissolved with 9 g/l normal saline into liquid with 0.4 g/ml of concentration, and the volume of gastric capacity was 0.67 ml/100 g. we chose a small dose of porcine serum (Thermo Fisher, 26250084) (0.5 ml/kg, twice a week), which is managed with intraperitoneal injection methods for modeling [35]. At the 18^th^ week of modeling, the sequence of miR-29b-3p was inserted into AAV expression vector (provided by Sangon Biotech Co., Ltd. Shanghai, China) and was injected into the tail vein, with an injection volume of 200 μl and an injection volume of 50 μl for each injection (reference: the *in vivo* transfection instructions). In the 22^nd^ week of modeling, 1% pentobarbital was applied to anesthetizing rats intraperitoneally. Then rats were killed by exsanguination, and the specimens were collected and forwarded to the later testing stage.

### XSSJS drug-contained serum intervention

Next, we randomly split the 20 Wistar rats into the control group and drug-contained serum group. XSSJS no-decoction granules were directly infused into the stomachs of Wistar rat (twice a day at a fixed time) for drug-contained serum group, with other conditions (eg. capacity, concentration) unaltered as indicated above, while the control group was established via gavage with the same volume of 9 g/l normal saline. In the seventh day of intragastric administration, 1% pentobarbital was applied to anesthetize rats intraperitoneally, and blood was taken from the posterior abdominal aorta in sterile environment, centrifuged at 2000 rpm (r = 13.5 cm) for 20 min, the serum was separated 56°C water bath for 30 min, 0.22 μm membrane filtration, and finally the FBS in HSC-T6 medium after miR-29b-3p transfection was replaced by XSSJS containing serum and cultured for 24 h.

### Real-time quantitative polymerase chain reaction analysis

TRIzol reagent was used to extract the complete RNA from cells, and then detected the integrity of RNA with 1% agarose electrophoresis via calculating with the ratio of A 260 nm/A280 nm, when the purity of RNA in 1.8–2.2, the cDNA was synthesized using a HyPure™ Molecular Biology Grade Water 5× All-In-One MasterMix (with AccuRT Genomic DNA Removal Kit, HyClone). After that, the cDNA was served as the substrate for real-time quantitative polymerase chain reaction (RT-qPCR) with EvaGreen Express 2× PCR MasterMix-No Dye (ABM Goodchina Inc.), the primer sequences used in RT-qPCR analysis are shown in Table 1. 2^−ΔΔ*C*_t_^ method makes the calculation for the relative levels of mRNAs, β-Actin is the reference gene. The whole progress of manipulation follows the manufacturer’s reagent instructions.

### Western blotting assays

The sodium dodecyl sulfate/polyacrylamide gel electrophoresis (SDS/PAGE) was prepared to separate from each group after treatment, the protein samples of liver tissue and cells (Wuhan Google Biotechnology Co., Ltd.), which would be subsequently transferred to 0.45-μm PVDF membrane (Millipore Company, MA, U.S.A.) according to different molecular weight of each protein at low temperature. After that, the cell membrane was incubated with the indicated primary antibody at 4°C overnight. The dilution ratio of primary antibody is 1:1000. Subsequently, the membranes were moved into the secondary antibody incubation box with a dilution ratio of 1:5000 for 60 min at room temperature, after which the protein expression was tested with ECL Kit (Hangzhou Lianke Biotechnology Co. Ltd, China). GAPDH was set as an internal reference. Chemiscope analysis software was used for gray analysis (Shanghai Qinxiang Scientific Instrument Co., Ltd), whose relative expression was calculated with respect to the protein of interest and GAPDH in the same sample and expressed graphically.

### Luciferase gene reporter assay

The mechanism of miR-29b-3p and VEGFA on the molecules via luciferase assay was confirmed. The target promoter fragment, cloned from genomic DNA using PCR technology, was inserted into luciferase reporter gene plasmid PGL3-BASIC by double digestion assay of restriction enzyme SacI and XbaI. Luciferase reporter plasmids including the 3′-UTR of VEGFA (called as VEGFA-WT) and the empty luciferase vector were purchased from the Shanghai Integrated Biotech Solutions Co.,Ltd. (Shanghai, China, IBS-01). Mutations were made at the predicted target sites between miR-29b-3p and VEGFA, therefore the name, VEGFA-MUT. HEK 293T cell were seeded in 96-well plates and co-transfected with the VEGFA-WT/VEGFA-MUT and mimic-NC/mimic-miR-29b-3p. Lipofectamine™ 2000 transfection reagent was applied for cell transfection based on the specification. After 48 h of the con-transfection with mimic-miR-29b-3p and VEGFA plasmids, the luciferase activity in each well was measured by using Luciferase reporter gene detection kit (shanghai Beyotime Biotechnology) (firefly luciferase was used as a reporter gene and sea kidney luciferase was used as an internal reference gene).

### Histopathology assay

The hepatic tissues containing fixed 4% concentration of paraformaldehyde were embedded in paraffin. The 5-μm sections were sliced and stained with Hematoxylin–Eosin (HE) and Masson. Slides were scanned and images were taken under an optical microscope (400×; Shanghai Qinxiang Scientific Instrument Co., Ltd. China, Chemiscope 6100).

### Immunofluorescence staining

The evenly distributed cells in the middle of the cover glass were marked with a histochemical circle, and then 50–100 μl working solution was added. After 0.5 h of incubation, cells were washed twice with PBS for 5 min each time. The tissue was uniformly covered with 3%BSA for 30 min, thereafter PBS was added to the cell well plate, entailing an incubation period overnight at 4°C. The cellular porous plates were processed by the decolorization shaker for three-times, 5 min each time. Shortly after a momentary drying, the tissue was covered with secondary antibody of the corresponding species in the histochemistry kit and incubated at room temperature for 1 h. The slide was placed in PBS, and then shaken on the decolorization shaker for three-times, 5 min each time. After the sections slightly dried, DAPI dying solution was dropped in the circle to redye the nuclei, and the cells were incubated at room temperature for 10 min in dark, and then sealed with anti-fluorescence quenching sealing tablets. The sections were observed under fluorescence microscope, from which images were collected. The immunofluorescent intensity ratio was calculated between the target protein and endogenous protein.

### Statistical analysis

The quantitative data represented as mean (M) ± standard deviation (SD). The comparisons of among-group data were analyzed by Student’s *t* test and one-way ANOVA. Conventional statistical significance metric is adopted (*P*<0.05). All the statistical analyses were carried out using SPSS 12.0. The histograms were drawn in GraphPad Prism 6.0.

## Results

### VEGFA was a target of miR-29b-3p in rat species

**Figure 2:**
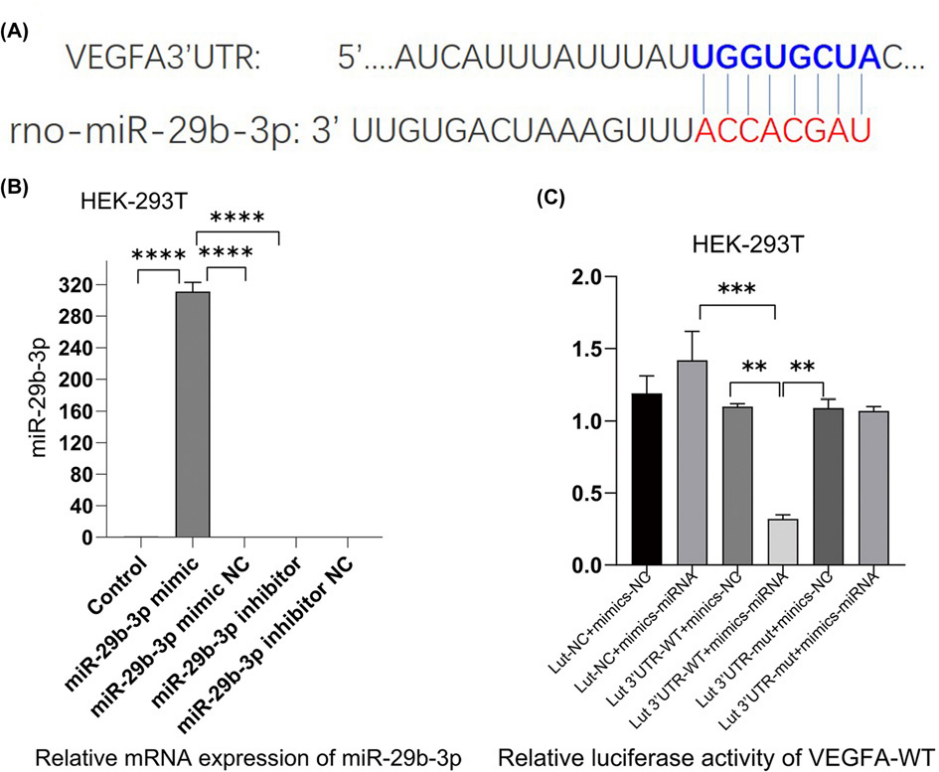
miR-29b-3p targeted VEGFA* in vitro* (**A**) The binding sites between miR-29b-3p and the 3′UTR of VEGFA. (**B**) RT-PCR tested the expression of miR-29b-3p following 24 h of cell transfection with miR-29b-3p mimics and miR-29b-3p inhibitor into HEK 293T cells. (**C**) A target gene of interest is transfected with a luc gene that expresses the enzyme luciferase at the target site, and the luciferase activity of VEGFA-WT was tested following 48 h of cell con-transfection (x ± s, *n*=3, **P<0.05, ***P<0.01, ****P<0.001).

We explored the relationship between miR-29b-3p and VEGFA *in vitro*. Figure 2A showed the binding sites between miR-29b-3p and the 3′UTR of VEGFA in rats. Compared with the miR-29b-3p mimic NC, transfection with miR-29b-3p mimic induced a clear increase in miR-29b-3p level in HEK-293T, whereas this effect was reversed by miR-29b-3p inhibitor (Figure 2B). The luciferase gene reporter assay showed that up-regulation (overexpression) of miR-29b-3p mimic reduced the luciferase activity of wet-VEGFA, nevertheless it was considered invalid when the binding sites of miR-29b-3p in the 3′UTR of VEGFA had changed due to mutation (Figure 2C). These findings confirmed that VEGFA was a target of miR-29b-3p in rat species.

### XXSJS intervention inhibited PA via promotion miR-29b-3p/VEGFA axis *in vitro*

#### Effect of XSSJS compound serum on proliferation of HSCT6 Cells

**Figure 3:**
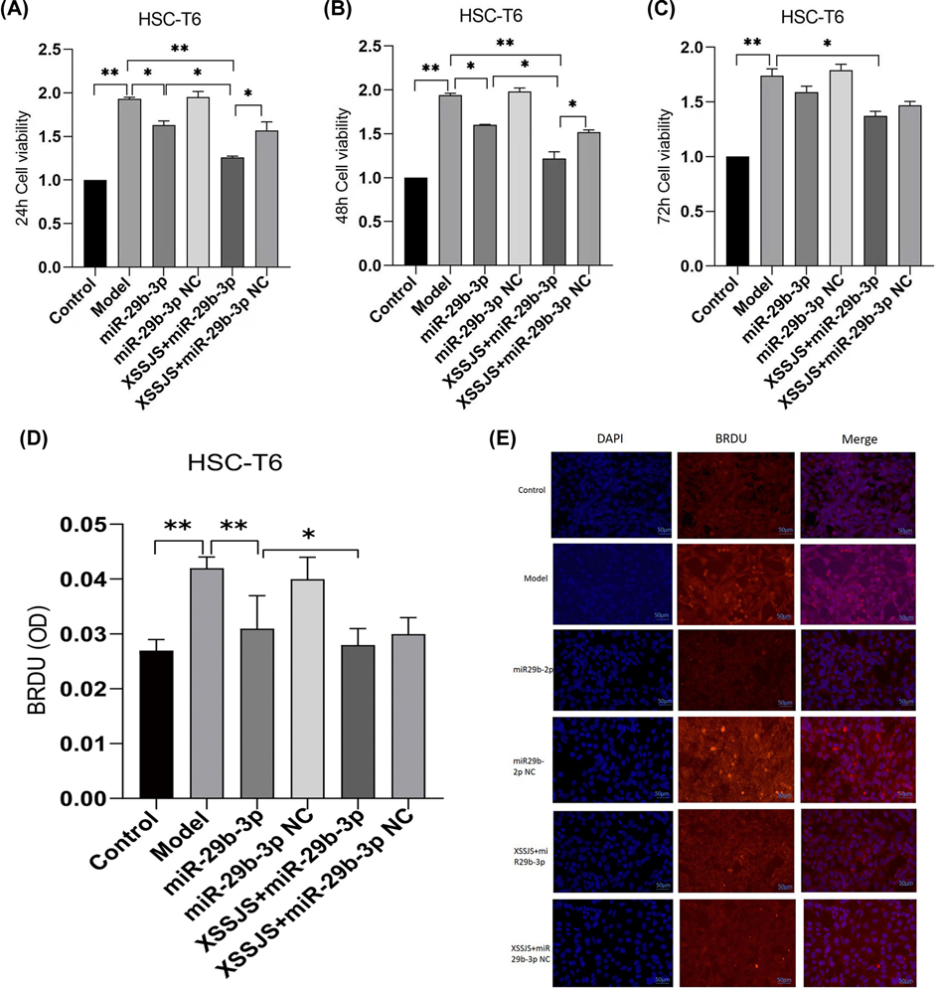
The cell proliferation was tested by using CCK8 and BrdU IF (**A–C**) The CCK8 proliferation assays (taken at 24, 48, 72 h, respectively) showed that XSSJS+miR-29b-3p better prevented the proliferation of HSC induced by TGF-β than XSSJS and miR-29b-3p at 24 and 48 h. There is little difference between their cell viability at 24 and 48 h, while the viability at 72 h is less effective than the viability at 24 or 48 h. (**D**) BrdU images showing that intervention with XSSJS+miR-29b-3p could more significantly decrease the number of BrdU-positive cells than XSSJS or miR-29b-3p administration. *Bars* = 50 μm. (**E**) Graphs of BrdU-positive ratios. All the experiments were repeated three times and represented as the means ± SEM (x ± s, *n*=3, *P<0.05, **P<0.01).

The CCK8 assay results (taken at 24, 48, 72 h, respectively) showed that HSC-T6 cells were pre-treated with TGF-β (20 ng/ml) followed by treatment with XSSJS compound serum for 24 h. The inhibitory effect of XSSJS and miR-29b-3p on cell proliferation were assessed. The results were found that, compared with the ineffectiveness on cell proliferation of the control group, cell has increased in the model group, while it decreased in the three groups of miR-29b-3p, XSSJS + miR-29b-3p and XSSJS + miR-29b-3p NC. Moreover, the decreased amount of XSSJS + miR-29b-3p group was more significant than the amount of the other two (Figure 3A–C), which indicated that XSSJS + miR-29b-3p could more significantly inhibit the HSC proliferation than using XSSJS or miR-29b-3p intervention alone. Cell proliferation was also assessed using BrdU staining, which showed that XSSJS + miR-29b-3p group could better suppress the proliferation of HSC compared with miR-29b-3p and XSSJS group alone. No significant difference in cell proliferation was observed between the miR-29b-3p and XSSJS + miR-29b-3p NC group (Figure 3D,E).

#### Effect of XSSJS compound serum on the expression of miR29b-3p, VEGFA, TGF-β, PDGF in HSCT6 cells

**Figure 4:**
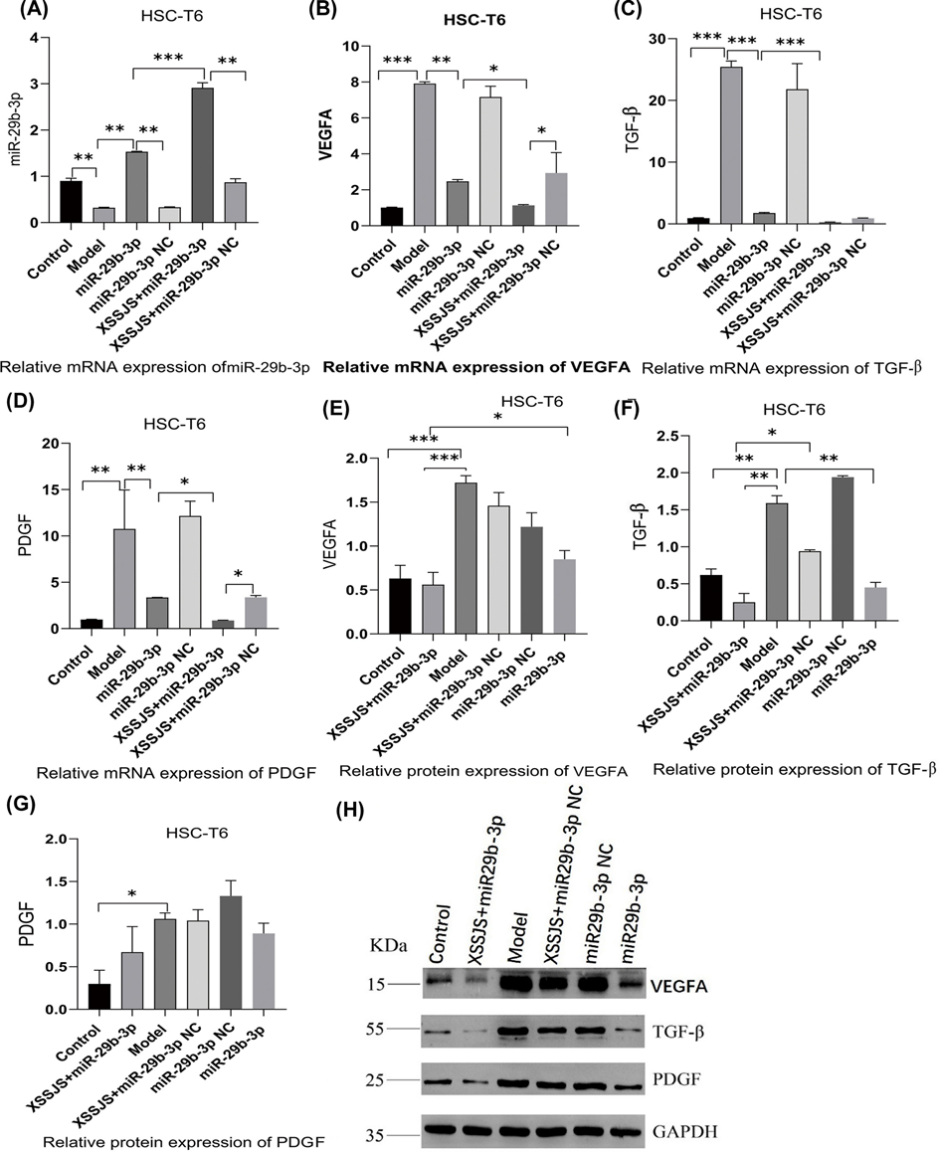
The expression of miR29b-3p, VEGFA, TGF-β, PDGF were tested following XSSJS compound serum intervention in HSCT6 cells (**A–D**) The mRNA levels of miR-29b-3p, VEGFA, PDGF, TGF-β were tested by RT-PCR. XSSJS intervention following miR-29b-3p transfection (XSSJS+miR-29b-3p group) more significantly promote the expression of miR-29b-3p and decrease the expression of VEGFA, PDGF, TGF-β. (**E–H**) The protein level of VEGFA, PDGF, TGF-β were tested by WB; the protein expression of VEGFA, TGF-β with XSSJS intervention following miR-29b-3p transfection (XSSJS+miR-29b-3p group) could be more significantly decreased than using XSSJS or miR-29b-3p alone (x ± s, *n*=3, *P<0.05, **P<0.01, ***P<0.001).

Subsequently, we explored whether miR-29b-3p/VEGFA axis was involved in XSSJS-mediated PA improvement *in vitro*. Firstly, XSSJS + miR-29b-3p promoted the miR-29b-3p expression more significantly than either miR-29b-3p or XSSJS+ miR-29b-3pNC group (Figure 4A). Accordingly, XSSJS + miR-29b-3p more significantly curbed VEGFA expression than miR-29b-3p or XSSJS (XSSJS+miR-29b-3p NC) (Figure 4B,E,H). Moreover, the level of other angiogenesis factors, such as TGF-β, PDGF, were significantly reduced by SXXJS+miR-29b-3p (Figure 4C,D,F–H). However, we found the relative mRNA expression of PDGF inconsistent with the protein expression (Figure 4D,G). Via analyzing the relevant literature and tests [36,37], this peculiar result of our experiment may either arise from the time lag in the expression profile between protein and mRNA, or the modification issue after the protein translation.

#### Protein expression of VEGFA, TGF, PDGF, CD31, CD34 in HSCT6 cells

**Figure 5:**
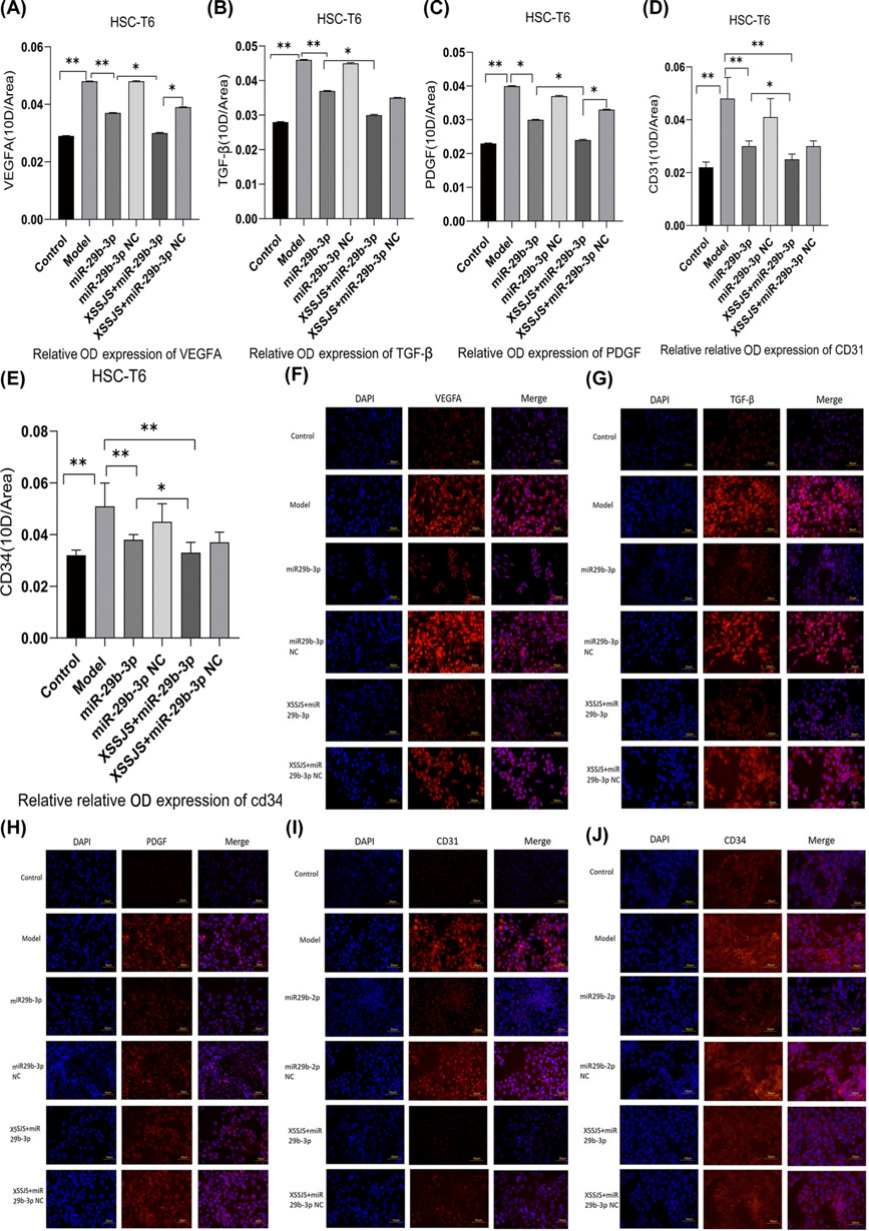
Protein expression of VEGFA, TGF, PDGF, CD31, CD34 in HSCT6 cells The protein expression of angiogenesis-related factors (**VEGFA, TGF, PDGF, CD31, CD34**) were tested by IF. (**A–E**) *Left column*, DAPI channel showing blue fluorescence, *middle column* biomarkers channel showing red fluorescence from the liposomes, *right column* is DAPI with biomarkers channels merged. *Bars* = 50 μm. The quantitative analysis of average fluorescence intensity obtained from ImageJ software (**F**–**J**) (400×, x ± s, *n*=3, *P<0.05, **P<0.01).

IF results indicated that XSSJS+miR-29b-3p is the most effective in decreasing the protein expression of VEGFA, as well as other pathological angiogenesis-related factors (TGF-β, PDGF, CD31, CD34) (Figure 5A–J). These results suggested that the XXSJS intervention with miR-29b-3p transfection could more significantly inhibit the protein expression of VEGFA in HF. In addition, XXSJS also could reduce the protein expression of other PA-related markers of liver fibrosis.

### XXSJS intervention inhibited PA via promotion miR-29b-3p/VEGFA axis *in vivo*

#### XSSJS intervention alleviated HF with reduced pathological course in HF rats

**Figure 6:**
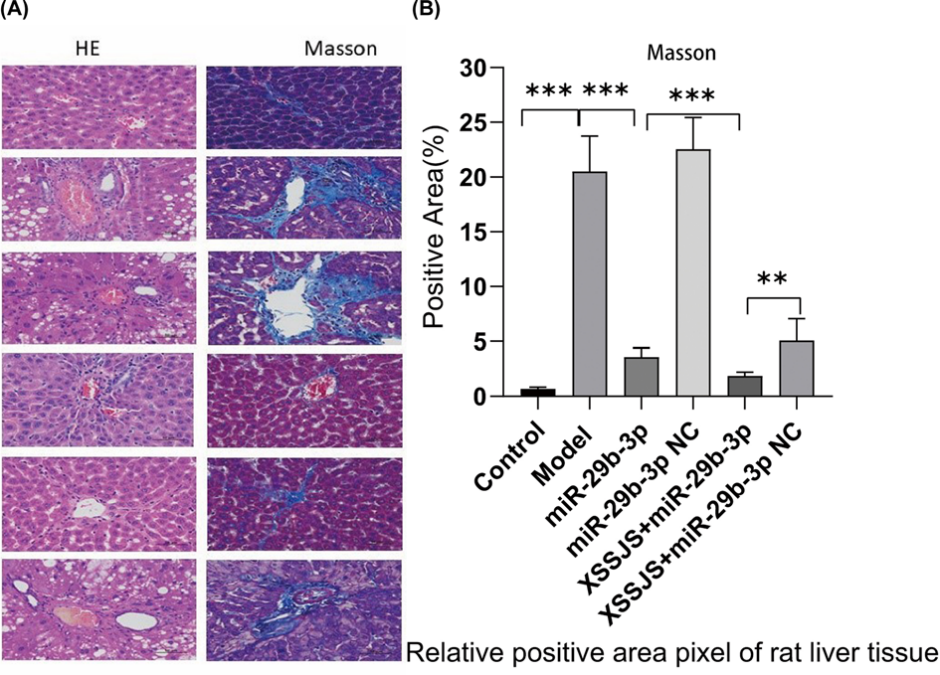
XSSJS intervention alleviated HF with reduced pathological course in HF rats (**A**) Comparison of pathological stains of each group after HE and Masson staining (400×). (**B**) Positive pixel area (%) of Masson are expressed (x ± s, *n*=3, **P<0.01, ***P<0.001).

Compared with the control group, the liver tissue appeared swollen and many of the cells contained vacuoles in the model group. Liver lobules were distorted by the irregular zones of parenchymal nodules that surrounded it. While the inflammatory cell infiltration was observed. All the above-mentioned pathological reactions are mitigated under the intervention of the following XSSJS, miR-29b-3p, and XSSJS+ miR-29b-3p, respectively, among those intervention the XSSJS+miR-29b-3p significantly reduced pathological response compared with the other groups (Figure 6A). The quantitative analysis of positive pixel area (%) in MASSON staining also exhibited the same effect (Figure 6B). Therefore, we can conclude that XSSJS intervention following miR-29b-3p transfection can reduce the degree of liver fibrosis, such as clear demarcation of hepatic lobules, insignificant proliferation of interlobular connective tissue, and less infiltration of inflammatory cells around hepatic sinusoids.

#### Effect of XSSJS on the expression of miR29b-3p, VEGFA, TGF-β, PDGF in HF rats

**Figure 7:**
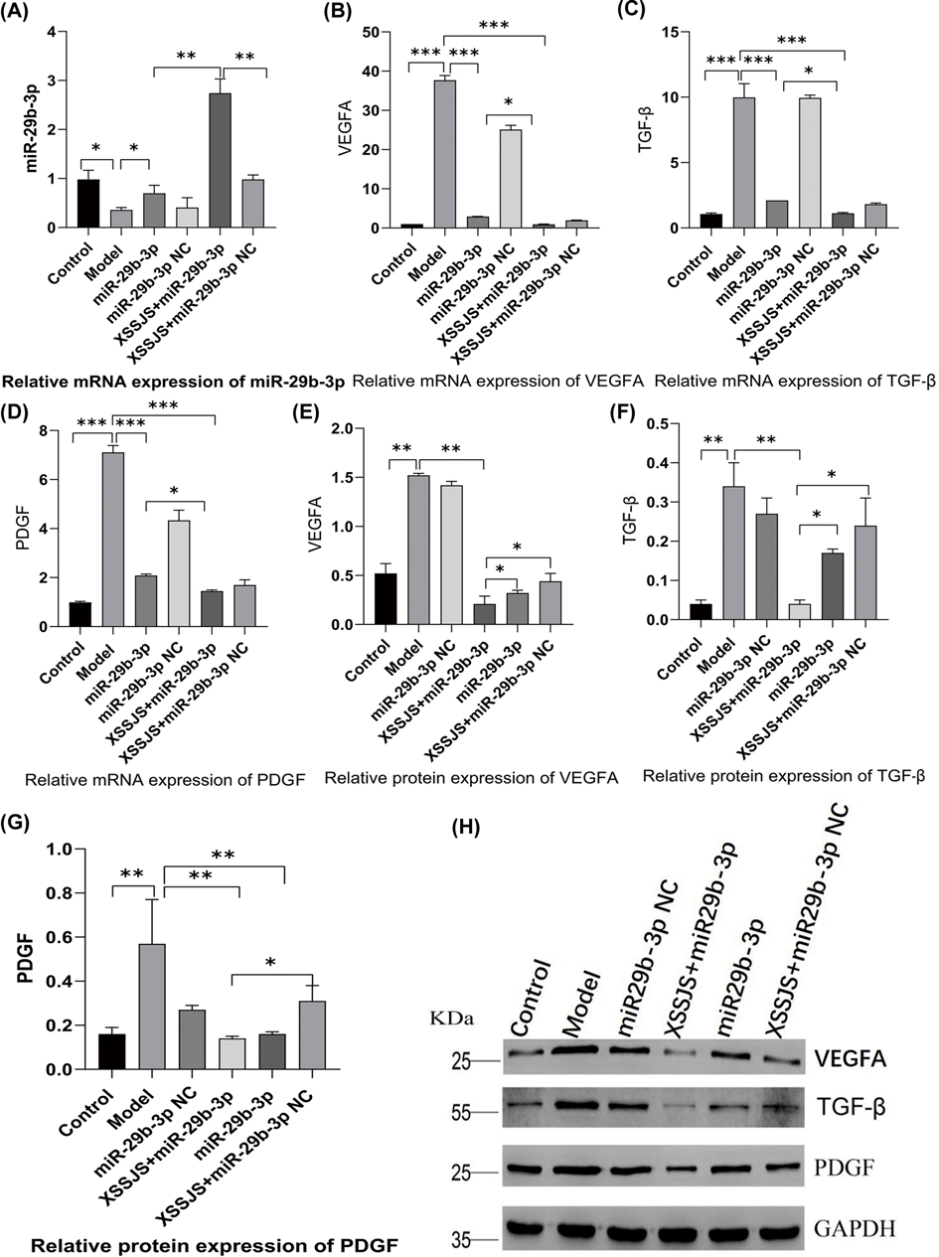
The expression of miR29b-3p, VEGFA, TGF-β, PDGF were tested following XSSJS intervention in HF rats (**A–D**) The levels of miR-29b-3p, VEGFA, PDGF, TGF-β mRNA were tested by using RT-PCR following XSSJS intervention in HF rats. (**E–H**) The protein levels of VEGFA, PDGF, TGF-β were tested by WB following XSSJS intervention in HF rats (x ± s, *n*=3, *P<0.05, **P<0.01, ***P<0.001).

In quest for further proof of miR-29b-3p/VEGFA axis being involved in XSSJS-mediated PA alleviation *in vivo*, we investigated the effectiveness of XSSJS intervention on the expression of miR-29b-3p and VEGFA in liver fibrosis rats. We found that the expression of VEGFA, PDGF, TGF-β remained relatively low in normal liver tissues, and VEGFA, PDGF, TGF-β were significantly up-regulated in HF rats. However, on the basis of miR-29b-3p AVV transfection *in vivo*, XSSJS treatment further increased the expression of miR-29b-3p in mRNA and reduced the expression values of VEGFA in mRNA and protein levels as well as in other pathological angiogenesis-related factors (TGF-β, PDGF) (Figure 7A–H). Those results suggested that XSSJS could promote the expression of miR-29b-3p, and its combination with miR-29b-3p has synergistic effect which could further reduce the level of VEGFA, TGF-β, PDGF than the separate use of either XSSJS or miR-29b-3p. Therefore, we can conclude that miR-29b-3p and VEGFA have implications in the role XSSJS played in mediating PA of HF.

#### The protein expression of VEGFA, TGF-β, PDGF, CD31, CD34 in HF rats

**Figure 8:**
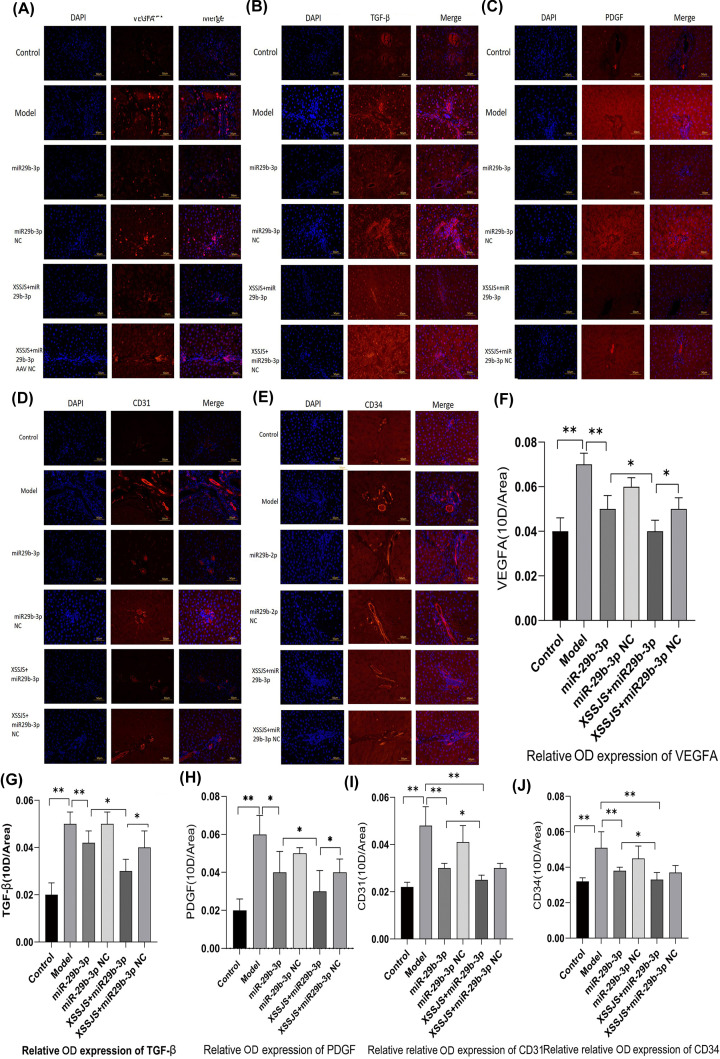
The protein expression of VEGFA, TGF-β, PDGF, CD31, CD34 in HF rats The protein expression of angiogenesis-related factors (VEGFA, TGF, PDGF, CD31, CD34) were tested by IF. (**A–E**) *Left column* DAPI channel showing blue fluorescence, *middle column* biomarkers channel showing red fluorescence from the liposomes, *right column* is DAPI with biomarkers channels merged. *Bars* = 50 μm. The quantitative analysis of average fluorescence intensity obtained from ImageJ software (**F–J**) (400×, x ± s, *n*=3, *P<0.05, **P<0.01).

IF imaging further confirmed that the relative expression of the target proteins (VEGFA, PDGF, TGF-β) were significantly decreased in the case of XSSJS treatment with miR-29b-3p AVV transfection (Figure 8A–C,F–H). Those results suggested that XSSJS could promote the expression of miR-29b-3p, and its combination with miR-29b-3p has synergistic effect which could further reduce the protein level of VEGFA, than the separate use of either XSSJS or miR-29b-3p, as well as reducing other pathological angiogenesis-related factors (TGF-β, PDGF). However, in the cases of CD31 and CD34, the aforementioned changes were not as significant, which invites future studies to investigate further (Figure 8D,E,I,J). In spite of this slight deficiency, we can still largely conclude that miR-29b-3p and VEGFA have implications in the role XSSJS played in mediating PA of HF.

## Discussion

HPA features the transformation of liver sinusoidal endothelial cells (LSECs) into the vascular type and contributes to the progression of cirrhosis and portal hypertension [38], VEGFA with a secreted growth factor containing signal peptides can specifically promote the mitosis of vascular endothelial cells and induce the occurrence of angiogenesis [39,40], which could regulate angiogenesis both in physiological and pathological conditions [41]. Previous studies in our team had proved that XSSJS could be used to reduce the density of liver microvessels via decreased levels of VEGF to inhibit fibrosis [23,24,26,42]. Given that the target relationship between miR-29b-3p and VEGFA had already been identified by dual-luciferase reporter assay in this study (Figure 3B,C), and that the mechanism for XSSJS to counter HF angiogenesis acts via miR-29b-3p/VEGFA axis, has been further demonstrated. Our experiment did take one step further than merely testing the VEGFA expression of the target gene, by forwarding the test to other relevant PA factors, such as CD31, CD34 [43], due to the traditional Chinese medication for being characterized by multiple targets [44] (XSSJS is a compound of multiple herb ingredients based on TCM and on the molecular level, its formulations typically feature multiple targets, multiple paths and multiple components).

Considering that the activation of HSCs is a key factor for HF [45], and TGF-β1 as a main profibrotic cytokines of HF [46–48] could induce VEGFA expression in HSCs [49], HSC-T6 cells were used in this study, which were activated by exposing the cells to TGF-β1. *In vitro*, the results demonstrated that XSSJS compound serum could up-regulate miR-29b-3p and decrease the expression of VEGFA and other pathological angiogenesis related factors (PDGF, TGF-β). The reasons are: (i) compared with the model group, it was found that cell viability was significantly decreased after XSSJS compound serum treatment following miR29b-3p transfection (Figure 3A–E); (ii) the expression of miR29b-3p was up-regulated and the production of VEGFA, PDGF, TGF-β were down-regulated after XSSJS compound serum intervention following miR-29b-3p transfection and its corresponding effectiveness gets the better of those of using XSSJS or miR29b-3p alone. *In vivo*, we observed that oral administration of XSSJS in rats with HF (once a day for 12 weeks) could promote the expression of miR-29b-3p and inhibit the expression of VEGFA, PDGF, and TGF-β in rats with liver fibrosis (Figure 7A–H). Furthermore, it could result in significant improvements of the conditions of liver injury, inflammation, fibrosis, and angiogenesis (Figure 6). In addition, the stability and homogeneity of animal models are essential to the study of HF, the present study was performed on a rat model induced by intraperitoneal injection of pig serum. The following two points: (i) the pattern of pig serum-induced immune HF is remarkably similar to that of the clinical pathogeny in human CLD [50,51]; (ii) unlike other chemical modeling methods, such as CCI4, the rat death rate is low and liver dysfunction is insignificant, could explain the reason why this approach has long since been extensively used and known as immune HF modeling [52]. Even so does the model conforms to the pathological characteristics of liver fibrosis in modern medicine, it does not accurately reflect the characteristics of collateral stasis involved in TCM syndrome typing. Therefore, in the future research, the model taking account of both the disease and syndrome will be the subject of study as to support the brand-new theory of TCM syndromes in more convincing manner, via which a prospective goal is to be attained, the combination of the TCM strengths and the multitarget, multichannel holistic treatment of Chinese medicine.

There is an issue in our study that needs some clarification: we found in those results concerning PA-related factors, the expression of mRNA of the *in-vivo* model denotes that the difference between XSSJS+miR29b-3p and XSSJS + miR29b-3p NC group does not emerge in a statistically significant fashion. Quite contrastingly, the protein result of the *in-vivo* model shows a significant difference between the XSSJS+miR29b-3p group and XSSJS+miR29b-3p NC group. Meanwhile, the results of the *in-vitro* models unanimously point to a significant difference between XSSJS+miR29b-3p group and XSSJS+miR29b-3p NC group, regardless it was with protein or mRNA (Figures 4 and 7). Therefore, via comprehensive overall data analysis, the aforementioned issue might be driven by factors such as the time lag between the tests on protein and nucleic acid, or the modification issue after the protein translation and potentially inappropriate handling during the experiment. Naturally, we will take this issue as one of the chief considerations in our future studies.

In addition, from a theoretical perspective, the different indicators that inhibit the same phenotype should increase or decrease homogeneously. However, CD31, CD34, as the most sensitive vessel endothelial markers, its abundance of expression fell short of the expected level (Figures 5D,E and 8I,J). Its reasons may have a tie-in to certain effect brought about by its intricate medical network (multitargeted), and the necessitated preparation for the direct verification experiment is underway. As a matter of course, these data of a kind out of the ordinary would merit more incisive studies in the future.

## Conclusion and future work

In the present study, the result demonstrated that XSSJS intervention could up-regulate miR-29b-3p and down-regulate VEGFA for both *in-vitro* and *in-vivo* cases. Therefore, we conclude that miR-29b-3p/VEGFA axis have implications in the role XSSJS played in combating PA of HF, which furnishes us the handle for explaining from the molecular level how XSSJS inhibits PA in the event of liver fibrosis. The PA test results of our experiment, especially regarding CD31, CD34, on angiogenesis had not been able to incisively reveal much detailed information due to the changes induced by using XSSJS with miR-29b being not as significant as other PA results. In the light of this, it comes to our decision that we will enter upon a second phase of exploration right on the back of our present one: first, we will introduce a bidirectional regulatory mechanism (activation and inhibition) to further determine the relationship between XSSJS and miR-29b-3p/VEGFA axis. Meanwhile, it is proved fact that the combination of the ‘medicines–targets’ network [53,54] and biological system network can reveal the complex Chinese medicine pharmacological mechanisms on the ‘multiple-targets’ level. Therefore, we will explore the pharmacological mechanisms of XSSJS for inhibiting PA following down this route. Subsequently, we will further explore the in-depth insight behind this phenomenon. Besides, studies on the direct mechanisms of XSSJS for tackling angiogenesis (e.g. three-dimensional assays of vascular morphogenesis) will also be covered in the future.

## Supplementary Material

Supplementary files

Supplementary materials

## Data Availability

All the relevant data are contained in the main article and its supplementary files.

## Abbreviations

AAV: adeno-associated virus
BrdU: bromodeoxyuridine
CCK8: cell-counting-kit-8
CD31: platelet endothelial cell adhesion molecule-1
CLD: chronic liver disease
DAPI: 4′,6-diamidino-2-phenylindole
FBS: fetal bovine serum
GAPDH: glyceraldehyde-3-phosphate dehydrogenase
HE: Hematoxylin–Eosin
HF: hepatic fibrosis
HPA: hepatic pathological angiogenesis
HSC-T6: hepatic stellate cell T6
IF: immunofluorescence
PDGF: platelet-derived growth factor
RT-qPCR: real-time quantitative polymerase chain reaction
TCM: traditional Chinese medicine
TGF-β: transforming growth factor-β
VEGFA: vascular endothelial growth factor
XSSJS: Xueshisanjia

## References

[1] Bataller R. and Brenner D.A. (2005) Liver fibrosis. J. Clin. Invest. 115, 209–218 10.1172/JCI24282 15690074 PMC546435

[2] Yang L. et al. (2014) Vascular endothelial growth factor promotes fibrosis resolution and repair in mice. Gastroenterology 146, 1339.e1–1350.e1 10.1053/j.gastro.2014.01.061 24503129 PMC4001704

[3] Sahin H. et al. (2012) Chemokine Cxcl9 attenuates liver fibrosis-associated angiogenesis in mice. Hepatology 55, 1610–1619 10.1002/hep.25545 22237831

[4] Keeley E.C., Mehrad B. and Strieter R.M. (2008) Chemokines as mediators of neovascularization. Arterioscler. Thromb. Vasc. Biol. 28, 1928–1936 10.1161/ATVBAHA.108.162925 18757292 PMC2735456

[5] Strieter R.M. et al. (2005) CXC chemokines in angiogenesis. Cytokine Growth Factor Rev. 16, 593–609 10.1016/j.cytogfr.2005.04.007 16046180

[6] Sulpice E. et al. (2004) Platelet factor 4 disrupts the intracellular signalling cascade induced by vascular endothelial growth factor by both KDR dependent and independent mechanisms. Eur. J. Biochem. 271, 3310–3318 10.1111/j.1432-1033.2004.04263.x 15291808

[7] Kitade M. et al. (2006) Leptin-mediated neovascularization is a prerequisite for progression of nonalcoholic steatohepatitis in rats. Hepatology 44, 983–991 10.1002/hep.21338 17006938

[8] Adya R. et al. (2008) Visfatin induces human endothelial VEGF and MMP-2/9 production via MAPK and PI3K/Akt signalling pathways: novel insights into visfatin-induced angiogenesis. Cardiovasc. Res. 78, 356–365 10.1093/cvr/cvm111 18093986

[9] Vizio B. et al. (2021) Cooperative role of thrombopoietin and vascular endothelial growth factor-A in the progression of liver cirrhosis to hepatocellular carcinoma. Int. J. Mol. Sci. 4, 1818 10.3390/ijms22041818 33673041 PMC7918121

[10] Salcedo X. et al. (2005) The potential of angiogenesis soluble markers in chronic hepatitis C. Hepatology 42, 696–701 10.1002/hep.20828 16104024

[11] Salcedo X. et al. (2005) Review article: angiogenesis soluble factors as liver disease markers. Aliment. Pharmacol. Ther. 22, 23–30 10.1111/j.1365-2036.2005.02532.x 15963076

[12] Friedman S.L. (1993) Seminars in medicine of the Beth Israel Hospital, Boston. The cellular basis of hepatic fibrosis. mechanisms and treatment strategies. N. Engl. J. Med. 328, 1828–1835 10.1056/NEJM199306243282508 8502273

[13] Lee K.S. et al. (1995) Activation of hepatic stellate cells by TGF alpha and collagen type I is mediated by oxidative stress through c-myb expression. J. Clin. Invest. 96, 2461–2468 10.1172/JCI118304 7593635 PMC185899

[14] Iwakiri Y. (2014) Pathophysiology of portal hypertension. Clin. Liver Dis. 18, 281–291 10.1016/j.cld.2013.12.001 24679494 PMC3971388

[15] Iwakiri Y. (2012) Endothelial dysfunction in the regulation of cirrhosis and portal hypertension. Liver Int. 32, 199–213 10.1111/j.1478-3231.2011.02579.x 21745318 PMC3676636

[16] Llovet J.M. et al. (2008) Sorafenib in advanced hepatocellular carcinoma. N. Engl. J. Med. 359, 378–390 10.1056/NEJMoa0708857 18650514

[17] Shah V.H. and Bruix J. (2009) Antiangiogenic therapy: not just for cancer anymore? Hepatology 49, 1066–1068 10.1002/hep.22872 19330868

[18] Siegel A.B. et al. (2008) Phase II trial evaluating the clinical and biologic effects of bevacizumab in unresectable hepatocellular carcinoma. J. Clin. Oncol. 26, 2992–2998 10.1200/JCO.2007.15.9947 18565886 PMC3635806

[19] Mu Y. et al. (2006) The inhibitory effect of the Xiayuxue decoction on liver fibrosis in rats during the progression period and its dialectical discussion. J. Tradit. Chin. Med. 47, 215–218

[20] Yao F., Lau P., Sun J. et al. (2018) Clinical study on the reversal of liver fibrosis and its regulation of immune function by “the healthy and soft liver blood flow side of the spleen”. Jiangsu Tradit. Chin. Med. 50, 42–44

[21] Lu J., Tan W., Zhao Z. et al. (2015) The anti-hepatic fibrosis mechanism of the complex component of fuzheng Huayu inhibits the rebirth of blood vessels. World Chin. Med. 2, 186–191

[22] Zhao P. (2011) Advances in the study of curcumin anti-angiogenic rebirth. Med. Clin. Res. 28, 1407–1409

[23] Zheng X., Hui Y., Yu S. et al. (2014) Effects of triamcinolone on the expression of HSC-T6 cells α-SMA, TIMP-1, and ROCK-I proteins. J. Tianjin Univ. Trad. Chin. Med. 6, 359–361

[24] Wang Ju, Based on the theory of “main passenger communication” the study on the effect of addition and subtraction of triacetate on the signaling pathway of angiogenesis in liver fibrosis rats. Doctoral Dissertation Chengdu University of Traditional Chinese Medicine https://kreader.cnki.net/Kreader/CatalogViewPage.aspxdbCode=CDFD&filename=1016045289.nh&tablename=CDFDLAST2016&compose=&first=1&uid=

[25] Li Y., Sun Y., Fenghua L. et al. (2013) The prevention and treatment effect of Huayu meidicine on liver fibrosis rats and their effect on the TGF-beta 1/Samd signaling pathway. The 25th National Conference on Chinese and Western Medicine Combined with Digestive Diseases https://d.wanfangdata.com.cn/conference/8256024

[26] Wang B., Kang Y., Xu Y. et al. (2016) Effects of XSSJS on liver microvascular density and VEGF-α expression in immun opastic fibrosis rats. Chinese J. Tradit. Chin. Med. 5, 1705–1709

[27] Roderburg C., Trautwein C. and Luedde T. (2011) MicroRNA-199a/b-3p: a new star in the liver microcosmos. Hepatology 54, 729–731 10.1002/hep.24456 21793019

[28] Tuomi T. et al. (2014) The many faces of diabetes: a disease with increasing heterogeneity. Lancet 383, 1084–1094 10.1016/S0140-6736(13)62219-9 24315621

[29] Sciarretta S., Volpe M. and Sadoshima J. (2014) Mammalian target of rapamycin signaling in cardiac physiology and disease. Circ. Res. 114, 549–564 10.1161/CIRCRESAHA.114.302022 24481845 PMC3995130

[30] Friedman S.L. et al. (1989) Maintenance of differentiated phenotype of cultured rat hepatic lipocytes by basement membrane matrix. J. Biol. Chem. 264, 10756–10762 10.1016/S0021-9258(18)81686-6 2732246

[31] Koyama Y. et al. (2016) New developments on the treatment of liver fibrosis. Dig. Dis. 34, 589–596 10.1159/000445269 27332862 PMC4961096

[32] Koyama Y. and Brenner D.A. (2015) New therapies for hepatic fibrosis. Clin. Res. Hepatol. Gastroenterol. 39, S75–S79 10.1016/j.clinre.2015.06.011 26206573 PMC4734896

[33] Dong P., Zheng J., Lu Z. et al. (2013) Experimental study of nano-shell polysaccharides carrying miR-29b transfected liver star cells. China J. Health Insp. 23, 332–334

[34] Li X., Yang H., Xiang Y. et al. (2014) Rat CTGF miRNA plasmid vector construction and the establishment of stable transfection of liver astrocyte cell lines. The 25th National Conference on Chinese and Western Medicine Combined with Digestive Diseaseshttps://d.wanfangdata.com.cn/conference/8717989

[35] Liu X., Hu Y., Hu Y. et al. (2004) Comparison of tissue pathology between carbon tetrachloride and serum liver fibrosis models of pigs. World J. Chin. Diges. 12, 1875–1879

[36] Wen J.D. et al. (2008) Following translation by single ribosomes one codon at a time. Nature 452, 598–603 10.1038/nature06716 18327250 PMC2556548

[37] Zheng Q. et al. (2010) Genome-wide double-stranded RNA sequencing reveals the functional significance of base-paired RNAs in Arabidopsis. PLoS Genet. 6, e1001141 10.1371/journal.pgen.1001141 20941385 PMC2947979

[38] Zhou W.C., Zhang Q.B. and Qiao L. (2014) Pathogenesis of liver cirrhosis. World J. Gastroenterol. 20, 7312–7324 10.3748/wjg.v20.i23.7312 24966602 PMC4064077

[39] Melincovici C.S. et al. (2018) Vascular endothelial growth factor (VEGF) - key factor in normal and pathological angiogenesis. Rom. J. Morphol. Embryol. 59, 455–467 30173249

[40] Ferrara N., Gerber H.P. and LeCouter J. (2003) The biology of VEGF and its receptors. Nat. Med. 9, 669–676 10.1038/nm0603-669 12778165

[41] Hoeben A. et al. (2004) Vascular endothelial growth factor and angiogenesis. Pharmacol. Rev. 56, 549–580 10.1124/pr.56.4.3 15602010

[42] Mu D., Wang B., Wang J. et al. (2017) Effects of addition and subtraction of triacetate on liver microvascular density and PDGF-B expression in liver fibrosis rats. Liaoning J. Tradit. Chin. Med. 7, 170–173 10.13192/j.issn.1000-1719.2017.07.057

[43] Ge Y. et al. (2013) Circulating CD31+ leukocyte frequency is associated with cardiovascular risk factors. Atherosclerosis 229, 228–233 10.1016/j.atherosclerosis.2013.04.017 23701996 PMC3984590

[44] Zhou S. et al. (2020) Deciphering the pharmacological mechanisms of taohe-chengqi decoction extract against renal fibrosis through integrating network pharmacology and experimental validation in vitro and in vivo. Front. Pharmacol. 11, 425 10.3389/fphar.2020.00425 32372953 PMC7176980

[45] Deleve L.D., Wang X. and Guo Y. (2008) Sinusoidal endothelial cells prevent rat stellate cell activation and promote reversion to quiescence. Hepatology 48, 920–930 10.1002/hep.22351 18613151 PMC2695448

[46] Inagaki Y. and Okazaki I. (2007) Emerging insights into Transforming growth factor beta Smad signal in hepatic fibrogenesis. Gut 56, 284–292 10.1136/gut.2005.088690 17303605 PMC1856752

[47] Saito D. et al. (2013) Transforming growth factor-β1 induces epithelial-mesenchymal transition and integrin α3β1-mediated cell migration of HSC-4 human squamous cell carcinoma cells through Slug. J. Biochem. 153, 303–315 10.1093/jb/mvs144 23248240

[48] Xu T. et al. (2016) NLRC5 regulates TGF-β1-induced proliferation and activation of hepatic stellate cells during hepatic fibrosis. Int. J. Biochem. Cell Biol. 70, 92–104 10.1016/j.biocel.2015.11.010 26592197

[49] Mitchell S.J. et al. (2011) Age-related pseudocapillarization of the liver sinusoidal endothelium impairs the hepatic clearance of acetaminophen in rats. J. Gerontol. A Biol. Sci. Med. Sci. 66, 400–408 10.1093/gerona/glq221 21300741 PMC3055277

[50] Huang Z. (1997) The serum-induced model of liver fibrosis and its mechanism in rats of heterogeneous animals. J. Shanghai Med. Univ. 24, 351–354

[51] Wu J. et al. (2009) Pig serum joint con a builds a model of liver fibrosis in Balb/c mice and evaluates sage. J. Anat. 32, 546–548

[52] Lin D. and Jin Z. (2006) Research and evaluation of commonly used animal models of liver fibrosis. Shizhen Natl. Med. 17, 487–488

[53] Chatr-Aryamontri A. et al. (2017) The BioGRID interaction database: 2017 update. Nucleic Acids Res. 45, D369–D379 10.1093/nar/gkw1102 27980099 PMC5210573

[54] Wang H. et al. (2020) A combined phytochemistry and network pharmacology approach to reveal the effective substances and mechanisms of Wei-Fu-Chun tablet in the treatment of precancerous lesions of gastric cancer. Front. Pharmacol. 11, 558471 10.3389/fphar.2020.558471 33381024 PMC7768900

