# Supplementary material for: XSSJS inhibits hepatic fibrosis by promoting the miR-29b-3p/VEGFA axis in vitro and in vivo
Source: Biosci Rep. 2022 Feb 25;42(2):BSR20212241. doi: 10.1042/BSR20212241 (PMC8881647; doi:10.1042/BSR20212241)
Supplement: Supplementary files [file BSR-2021-2241_supp.pdf]

Supplementary Material 2 Dataset, The cell proliferation was tested by using CCK8 and BRDU immunofluorescence

Supplementary Material 3 Dataset, The expression of miR29b-3p, VEGFA, TGF-β, PDGF were tested following XSSJS Compound Serum intervention in HSCT6 Cells

Supplementary Material 4 Dataset, The expression of miR29b-3p, VEGFA, TGF-β, PDGF were tested following XSSJS intervention in HF rats

Supplementary Material 5 Dataset, XSSJS intervention alleviated hepatic fibrosis with reduced pathological course in HF rats

Supplementary Material 6 The expression of VEGFA, TGF-β, PDGF were tested following XSSJS Compound Serum intervention in HSCT6 Cells

Supplementary Material 19, The expression of VEGFA, TGF-β, PDGF were tested following XSSJS intervention in HF rats

Supplementary Materials 7-18 and 20-31 are raw Western blots.
